# Some Reports of the Work of 1919

**Published:** 1920-03-27

**Authors:** 


### HOSPITAL FINANCES.

### Some Reports of the Work of 1919.

BLACKBURN AND EAST LANCASHIRE ROYAL INFIRMARY.—The total income for 1919 amounted to £20,740, and, as the expenditure under all heads amounted to £19,229, there was sufficient balance to clear up the deficiency in the previous year. Annual subscriptions increased from £1,624 in 1917 to £2,399 in 1919. This hospital is evidently quite alive to the benefit of self-help.

CITY OF LONDON MATERNITY HOSPITAL.—The total income was £12,557 and the expenditure £12,411. There were no legacies. The payments of patients in private wards amounted to £1,378.

The DEWSBURY AND DISTRICT GENERAL INFIRMARY, with a modest budget of about £7,000, finished the year slightly better off than at the commencement. The infirmary has now a comfortable reserve fund amounting to £30,000.

GLASGOW ROYAL INFIRMARY.—Here the financial outlook is not so bright as in many other places. The ordinary revenue was £52,802 and the ordinary expenditure £97,527. Legacies and large donations amounting to £24,924 were received, but a deficiency of £22,897 had to be carried to capital account.

KING EDWARD VII. HOSPITAL, WINDSOR.—The annual report makes use of Sir Henry Burdett's manifesto in the *Times* in approaching its supporters. What has been done at Nottingham, Bedford, Warrington, and elsewhere may very well be done also, it is argued, in Windsor —a much smaller city.

THE MILDMAY MISSION HOSPITAL, BETHNAL GREEN.—Subscriptions and donations last year are above the total of 1918 by £1,115. The total income was £6,107 and the total expenditure £6,755.

The preliminary report of the MANCHESTER ROYAL INFIRMARY is dated January 27, and contains full statistics and accounts. The total income for the past year was £98,142 and the expenditure £95,518. In addition a sum of £6,250 was carried direct to the balance sheet for endowment. As nearly £8,000 was spent under "extraordinary expenditure," this sum coming, of course, from current income, the hospital may very well be said to have had a good year.

ANCOATS HOSPITAL, in the same neighbourhood, has not done quite so well. The total income of the hospital and convalescent home was £20,273 and the total expenditure £24,379. But legacies, which by provincial fashion are not included in the income and expenditure account, amounted to £4,874, and £1,500 was given for endowment.

THE NORTHAMPTON GENERAL HOSPITAL.—The balance to the good in the 1918 accounts almost met the deficiency in 1919. Total ordinary income, £24,942; total ordinary expenditure, £28,227. But a special donation and legacies together amounting to £932 are carried direct to an investment account.

PONTYPOOL AND DISTRICT HOSPITAL.—This report, as usual, is very nicely printed, arranged, and illustrated. Total income, £6,471; total expenditure, £5,576.

ROYAL DEVON AND EXETER HOSPITAL.—The total income, including legacies received (£778), was £19,580; the total expenditure £20,182. The nursing institution revenue was £1,311 and contributions on behalf of patients amounted to £6,246.

ROYAL SUSSEX COUNTY HOSPITAL, BRIGHTON.—Total income (including £2,348 from legacies), £23,188; total expenditure, £28,608. The reception of patients able to contribute towards the cost of maintenance is under consideration.

ST. ANDREW'S HOSPITAL, DOLLIS HILL.—This hospital is largely for the benefit of contributing patients, but is conducted on a charitable basis. There was a deficiency on the year's working of £965.

WEST OF ENGLAND EYE INFIRMARY, EXETER.—The financial year runs from Michaelmas to Michaelmas. Income, £2,082; expenditure, £1,706. A small ward has been set aside for newly-born babies suffering from infectious eye disease; it is intended that their mothers shall be accommodated with the babies.

The financial year of SALOP ROYAL INFIRMARY ends on June 30. An effort to raise 100,000 shillings to pay off debt is, we are informed by the Secretary, Mr. Alfred Sugden, doing particularly well. There was a deficit on the working for the twelve months under review of £6,918.

